# Reduced birth weight, cleft palate and preputial abnormalities in a cloned dog

**DOI:** 10.1186/1751-0147-56-18

**Published:** 2014-03-26

**Authors:** Min Jung Kim, Hyun Ju Oh, Geon A Kim, Young Kwang Jo, Jin Choi, Hye Jin Kim, Hee Yeon Choi, Hyun Wook Kim, Min Cheol Choi, Byeong Chun Lee

**Affiliations:** 1 Department of Theriogenology and Biotechnology, College of Veterinary Medicine, Seoul National University, 1 Gwanak-ro, Gwanak-gu, Seoul 151-742, Republic of Korea; 2 Haemaru Referral Animal Hospital, 319 Beon-gil, Hwangsaeul-ro, Bundang-gu, Seongnam, Gyeonggi 463-824, Republic of Korea; 3 Department of Veterinary Medical Imaging, College of Veterinary Medicine, Seoul National University, 1 Gwanak-ro, Gwanak-gu, Seoul 151-742, Republic of Korea

**Keywords:** Developmental abnormality, Cleft palate, Penile frenulum, Preputial closure, Persistent penile extrusion, Cloning, Dog

## Abstract

The aim of the present study was to report a novel developmental abnormality in a cloned dog. A fibroblast cell line was established from an 8-year-old male German shepherd dog. *In vivo* matured oocytes were retrieved from a large breed dog, and the nucleus was removed from each oocyte. A donor cell was injected into an enucleated oocyte, and the oocyte-cell couplet was fused electrically. After chemical activation, the resulting embryos were transferred into a naturally estrus-synchronized recipient dog, and two cloned pups were delivered by Cesarean section 60 days later. One cloned pup (Clone 1) was healthy, but the other (Clone 2) had a birth weight of only 320 g and cleft palate, failure of preputial closure at the ventral distal part, and persistent penile frenulum. Clone 2 was raised by stomach feeding until Day 40 after birth, where palatoplasty was performed. The abnormalities in external genitalia in Clone 2 resulted in persistent penile extrusion that was surgically corrected. This complex developmental abnormality has not been reported in dogs previously.

## Background

Concurrent occurrence of congenital defects consisting of both cleft palate and abnormal external genitalia in dogs is rarely reported. Cleft palate/lip is one of the most common craniofacial congenital defects in humans (about 1/1000 infants) [1] and dogs [2,3]. It may result from incomplete fusion of the primary palate (lip and premaxilla) and secondary palate (hard and soft palates) during embryogenesis caused by genetic factors as well as environmental factors such as poor nutrition, drugs and teratogens, including virus [1,4]. Hypospadia is the most common congenital penile anomalies in humans [5] and dogs [6]. It develops due to abnormal fusion of the urogenital folds and ventral body wall [7,8]. Persistence of the penile frenulum, a thin band of connective tissue linking the ventral glans penis to the prepuce, has been rarely reported in dogs. It can be caused by failure of splitting of the balanopreputial fold, which is under androgenic control [9], and is observed with [10] or without [11] hypospadia. A retrospective study in humans revealed that more than 90% of male infants with a congenital penile anomaly exhibit only a single type [5].

Although clones and their nuclear donor animal have the same genome, developmental abnormalities have been reported in cloned mammals. Large offspring syndrome is one of the most frequently observed anomalies in cloned bovine offspring, characterized by fetal and placental overgrowth [12,13]. Recently, clones with congenital defects including immunodeficiency, abnormal knuckle, or several organ pathologies were also reported in cattle [14]. However, until now, developmental anomalies have been seldom reported in cloned dogs. Therefore, the aim of the present study was to report a complex of developmental abnormalities in a cloned dog.

## Case presentation

Mixed-breed dogs, aged between 1 and 7 years and weighing 20 to 35 kg, were used as oocyte donors and embryo transfer recipients. They were housed in separate indoor facilities and managed following a standard procedure established by the Committee for Accreditation of Laboratory Animal Care and according to the Guideline for the Care and Use of Laboratory Animals of Seoul National University. The experiment was approved by Institutional Animal Care and Use Committees of Seoul National University (approval number is SNU-121123-13).

Cloning procedures were done as previously reported [15]. In short, *in vivo* matured oocytes were retrieved by flushing oviducts about 72 h after ovulation and cumulus cells were removed by repeated pipetting in 0.1% (w/v) hyaluronidase in Hepes-buffered TCM-199 (Invitrogen, Carlsbad, CA, USA) supplemented with 2 mM NaHCO3, 5 mg/mL BSA (Invitrogen) and 12.9 μM kanamycin. Nuclear materials were removed from an oocyte, and a donor cell derived from an 8-year-old male German shepherd dog was injected into the enucleated oocyte. The oocyte-cell couplet was fused with two pulses of direct current (72 V for 15 μs) using an Electro-Cell Fusion apparatus (NEPA GENE, Chiba, Japan), then activated chemically. A total of 74 cloned embryos were transferred into 5 recipients. Pregnancy diagnosis by ultrasonography was performed at least 28 days after the embryo transfer and revealed one pregnancy. Two cloned German shepherd dog puppies were delivered from one recipient by Cesarean section 60 days after the embryo transfer; microsatellite analysis showed their genetic identity with the cell donor dog (Table 1).

**Table 1:** Microsatellite analysis of cell donor, oocyte donors and cloned dogs

**Sample ID**	**Marker**									
	**FH**	**FH**	**FH**	**FH**	**FH**	**FH**	**FH**	**FH**	**FH**	**FH**
	**2537**	**3027**	**3116**	**3372**	**3381**	**3399**	**1014**	**2097**	**2584**	**2712**
Cell donor	146	212	190	146	298	234	246	280	309	182
	/164	/212	/190	/154	/310	/250	/266	/284	/309	/184
Oocyte donor 1	164	216	190	146	282	262	250	276	301	180
	/164	/216	/192	/150	/286	/262	/250	/276	/301	/184
Oocyte donor 2	164	212	190	146	300	246	246	272	301	178
	/168	/212	/190	/154	/304	/254	/266	/284	/305	/182
Clone 1	146	212	190	146	298	234	246	280	309	182
	/164	/212	/190	/154	/310	/250	/266	/284	/309	/184
Clone 2	146	212	190	146	298	234	246	280	309	182
	/164	/212	/190	/154	/310	/250	/266	/284	/309	/184

**Figure 1:**
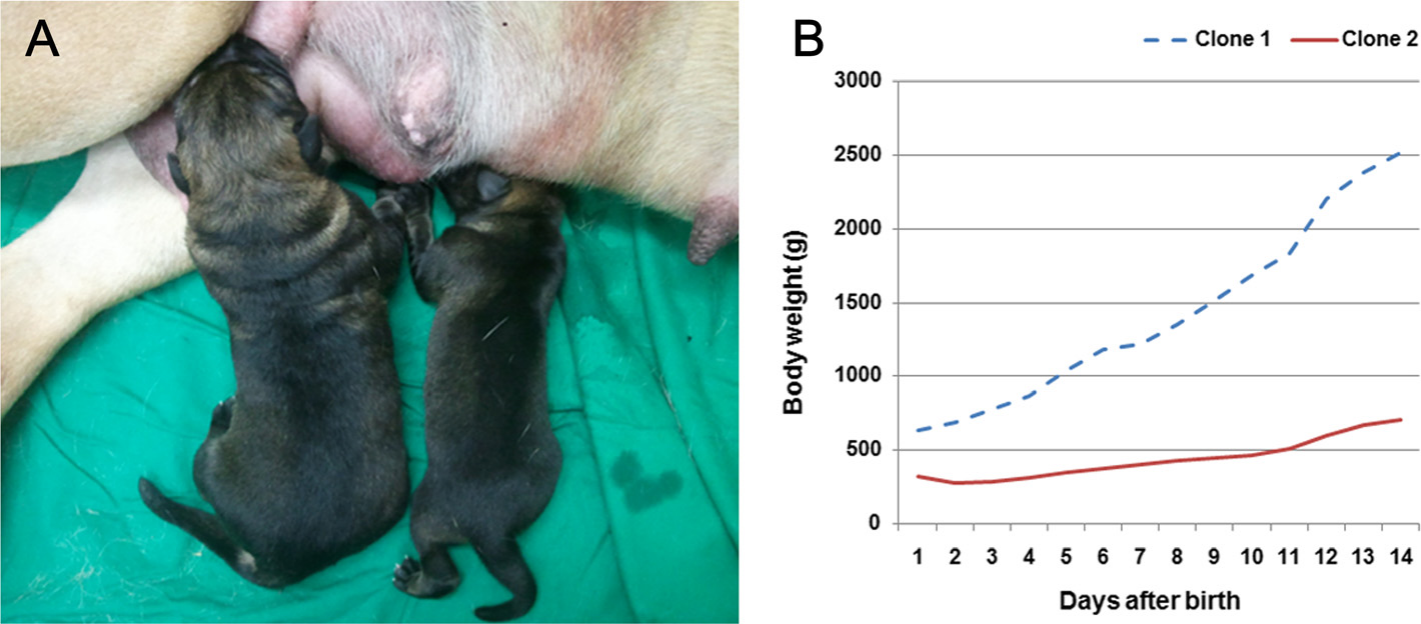
**Phenotypes and body weight increase chart of Clone 1 and Clone 2 puppies. (A)** Clone 1 (left) and Clone 2 (right) at Day 1 after birth. **(B)** Increase of body weight of Clone 1 (dotted) and Clone 2 (full line) during the first 2 weeks after birth.

**Figure 2:**
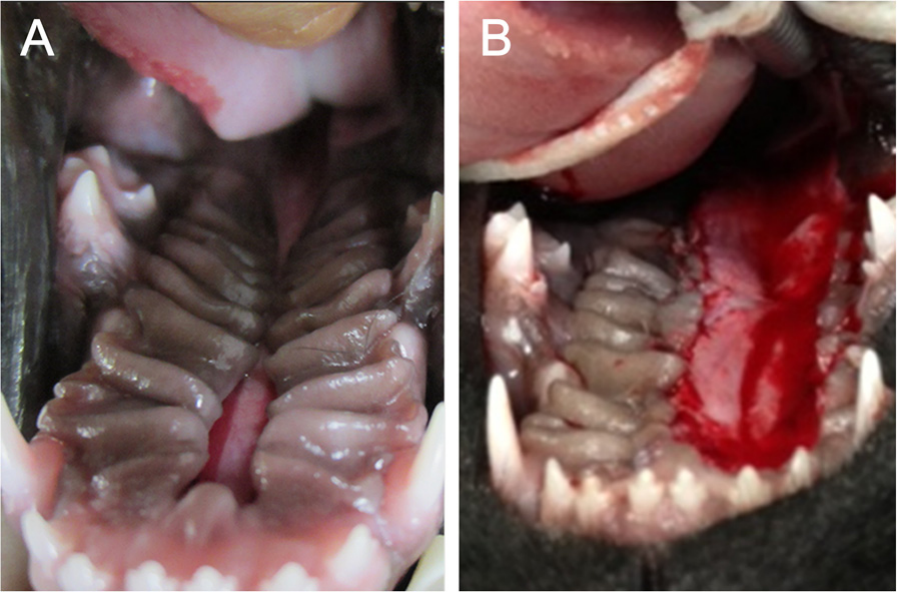
**Cleft palate of Clone 2. (A)** Cleft palate of Clone 2 at Day 35 after birth. **(B)** Palatoplasty of Clone 2 at Day 40 after birth.

**Figure 3:**
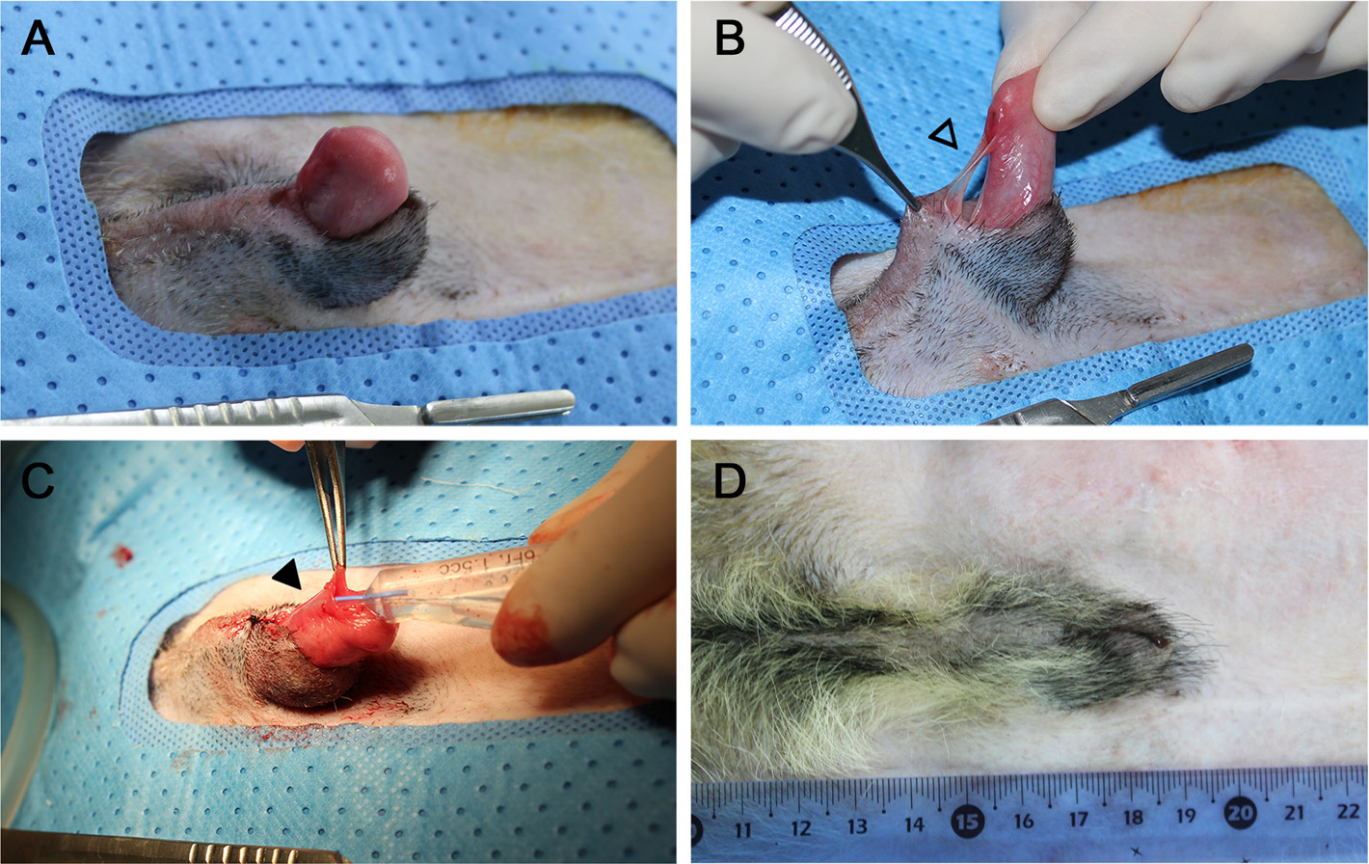
**Abnormal external genitalia of Clone 2 at Day 125 after birth. (A)** Persistent extrusion of penis without enlargement. **(B)** Abnormal connection between ventral distal end of penis and preputial mucosa (open arrow head). **(C)** Superficially opposed urethral opening and easily visualized urethral catheter (filled arrow head). **(D)** Completely healed prepuce 28 days after the corrective surgery.

The birth weight of Clone 1 (640 g) was twice that of Clone 2 (320 g) (Figure 1, A). While Clone 1 showed no phenotypic anomalies, Clone 2 had defects of the hard and soft palate (Figure 2, A) and penile extrusion (Figure 3, A). Thus, Clone 1 was fed and cared for by the embryo transfer recipient dog, but Clone 2 was fed milk replacement *via* stomach intubation and cared for by veterinarians. At 2 weeks of age, while Clone 1 reached 2,520 g body weight, Clone 2’s body weight was 710 g (Figure 1, B).

Due to anatomical limitations and limited tissue availability, palatoplasty in Clone 2 was performed on Day 40 after birth. Palatoplasty was performed by overlapping flap for repair of a cleft of the hard palate, and a medially positioned flap for repair of a cleft of the soft palate (Figure 2, B). Cefazolin 20 mg/kg (Hankook Korus Pharm Co., LTD., Seoul, Korea), tramadol 1 mg/kg (Samsung Pharm Ind. Co., LTD., Seoul, Korea), and amoxicillin–clavamox 13.75 mg/kg (Kuhnil Pharm Co., LTD., Chungnam, Korea) was given orally to the cloned dog for 2 weeks as post-operative medication.

Surgical correction of the developmental penile anomalies in Clone 2 was performed around 4 months of age. The penile extrusion was a result of persistent penile frenulum and failure of preputial closure in the ventral distal part (Figure 3, B). In addition, the opening of the urethra was continuous with the penile frenulum, and consequently the urethra was superficially positioned as the inserted urethral catheter could be seen (Figure 3, C). The penile frenulum was cut after confirming the position of the urethra by catheterization. The marginal skin region of the preputial failure was excised and the skin was closed by continuous subcutaneous suturing followed by skin apposition with simple interrupted sutures. Cephalexine 30 mg/kg BID, tramadol 2 mg/kg BID and streptokinase 0.3 mg/kg BID were administered for 7 days as post-operative medication. It was confirmed 1 month after the operation that the developmental penile anomalies in Clone 2 were corrected (Figure 3, D).

There is great concern about the health of clones, but until now, only three publications have reported developmental anomalies in cloned dogs. The first anomaly reported in cloned dogs was placentomegaly. Significantly higher placental weights were observed in the cloned group (135 **±** 10.1 g, n = 2), which used donor cells in confluency, compared with placentas in the natural breeding group (60 **±** 7.1 g, n = 4) [16], and the cloned pups were either delivered stillborn or died within a few days after birth [16], which might be the adverse effects of placental abnormalities [17]. A second study reported a transgenic cloned dog born with a malformation, but details were not described [18]. The third report described defects in the anterior abdominal wall, and increased heart and liver sizes, muscle mass and macroglossia in 12 deceased cloned dogs that died just prior to delivery or that died with dyspnea shortly after birth [19].

Cleft palate is a common congenital defect in dogs, and a puppy with this condition can suffer malnutrition, aspiration pneumonia or even death, if untreated. Although Clone 2 was weak born with a 320 g body weight, stomach feeding was effectively applied to Clone 2 until 2 weeks of age (710 g). Persistent penile extrusion in dogs often results from penile erection with [20] or without sexual behavior [21,22], but Clone 2 was born with persistent penile extrusion. Also, the penile frenulum in a dog usually does not accompany penile extrusion, and the failure of preputial closure at the ventral distal region in Clone 2 is not previously reported in the literature. Interestingly, a superficial position of the urethral opening was also found in Clone 2. Since congenital defects in both the craniofacial region and external genitalia develop from the ectoderm [4,23], these anomalies can be associated as seen in the Schilbach‒Rott syndrome, which is characterized by submucosal cleft palate, hypospadia and ocular hypotelorism in males [24]. On the other hand, they may develop independently because fusion processes of the secondary palate in craniofacial region are completed by the 10th week of embryogenesis [4], and desquamation of the epithelial fusion of prepuce usually occurs after birth [25]. The cause of this defect remains unknown. Further studies on genetic or epigenetic factors using the donor and the two cloned dogs described here would contribute to finding the etiology of these anomalies.

## Conclusions

To the authors’ knowledge, this is the first report of complex developmental abnormalities in the craniofacial region and external genitalia of a dog. Although the puppy was a clone, it remains hypothetical if the defect is associated with this. To determine the cause of this complex developmental anomaly, further studies on genetic or epigenetic factors are needed.

## Competing interests

The authors declare that they have no competing interests.

## Authors’ contributions

MJK participated in production of the cloned dogs, surgical correction of penile anomalies and drafting the manuscript. HJO participated in production of the cloned dogs and in revising the manuscript. GAK participated in production of the cloned dogs, surgical correction of penile anomalies and revising the manuscript. YKJ participated in production of the cloned dogs and surgical correction of penile anomalies. JC participated in production of the cloned dogs. HYC participated in surgical correction of the cleft palate. HJK participated in post-operative care and revising the manuscript. HWK participated in post-operative care and revising the manuscript. MCC participated in diagnosis of the cloned dog and revising the manuscript. BCL participated in production of the cloned dogs and drafting and revising the manuscript. All authors read and approved the final manuscript.
